# Recurrent midgut volvulus in an 83-year-old female

**DOI:** 10.1093/jscr/rjab298

**Published:** 2021-07-23

**Authors:** Lorna Kang, Brandon Larson, Shelly Barker, Truong Ma

**Affiliations:** Department of General Surgery, Summa Health System, Akron, OH, USA; Department of General Surgery, Summa Health System, Akron, OH, USA; Department of General Surgery, Summa Health System, Akron, OH, USA; Department of Colon and Rectal Surgery, Summa Health System Akron, OH, USA

## Abstract

We present a rare case of recurrent primary midgut volvulus in an elderly female with an interesting intraoperative finding of an abnormally elongated small bowel mesentery. This patient presented with symptoms of obstruction, including nausea, vomiting and obstipation, similar to previous episodes of volvulus for which she underwent exploratory laparotomies and reduction of the volvulus. We describe a novel use for enteropexy in which we effectively shortened the small bowel mesentery in an effort to eliminate the source of recurrent volvulus. The patient’s post-operative course was complicated by prolonged ileus requiring total parenteral nutrition. However, she had not developed signs or symptoms of bowel ischemia or recurrent volvulus at the time of this writing. Our findings suggest that enteropexy is an effective technique for preventing recurrent midgut volvulus primarily caused by abnormally elongated mesentery.

## CASE DESCRIPTION

**Figure 1:**
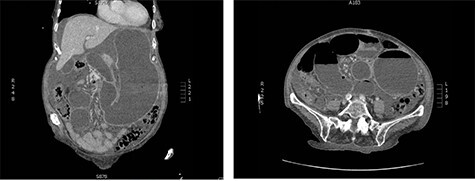
CT abdomen and pelvis on presentation; swirling of the SMA, markedly distended stomach and small intestine are noted.

An 83-year-old female presented to the emergency room with epigastric abdominal pain, nausea and vomiting for 1 day. She has a history of recurrent midgut volvulus for which she underwent two exploratory laparotomies in the prior 2 months, most recently, 3 weeks prior. In those operations, the patient was found to have an abnormally elongated small bowel mesentery which was torsed in a clockwise fashion. The mesentery was detorsed in both operations and no bowel resections nor pexy were performed. The operations were done open due to significant bowel distension and concern for potential bowel injury with a laparoscopic approach. She was recovering well from prior detorsion when she developed sudden onset nausea and vomiting 1 day prior to presentation. She also had not passed any flatus or bowel movements for the past 2 days. The patient’s past medical history was significant for Crohn’s disease, hypothyroidism and hypertension. She was recently diagnosed with Crohn’s but had not yet initiated treatment for it. She had no prior abdominal surgical history other than the recent exploratory operations. Computed tomography (CT) abdomen/pelvis obtained in the emergency room demonstrated gastric distension, swirling of the small bowel mesentery and ascites, concerning for small bowel obstruction secondary to recurrent midgut volvulus (Fig. 1). A nasogastric tube was inserted for decompression and the patient was emergently taken to the operating room for exploration and reduction of the volvulus.

## OPERATIVE DISCUSSION

**Figure 2:**
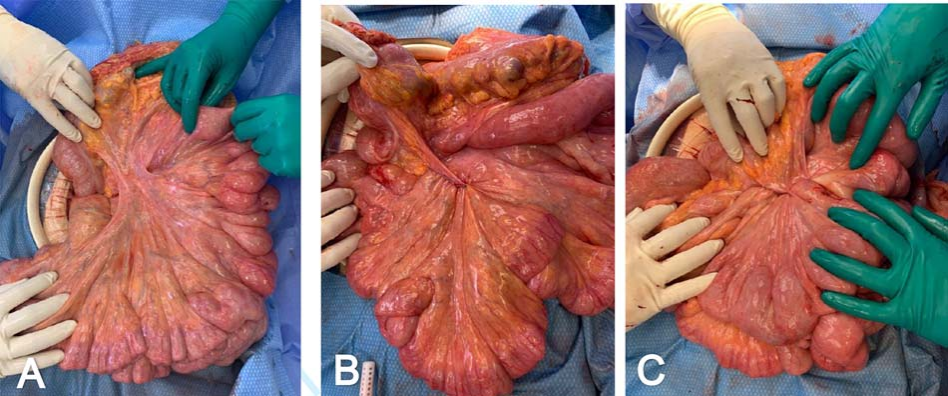
(**A**) Upon evisceration of the small bowel, abnormally elongated mesentary was noted; (**B**) small bowel mesentery pexy to the mesocolon is performed medial to lateral; (**C**) upon completion of the pexy, the small bowel mesentery is shortened and thickened.

The previous midline incision was opened. Dense adhesions were taken down sharply. There were loops of jejunum adhered to the abdominal wall which served as a point of volvulization, so this was reduced. Again, the abnormally long and narrow small bowel mesentery was noted (Fig. 2). There was no malrotation noted. The entire small bowel was run and appeared viable. A small segment of the mid-jejunum was resected due to an enterotomy and a side-to-side, functional end-to-end anastomosis was performed with an Endo GIA stapler. Attention was turned to the small bowel mesentery. We used 3-0 Ethibond suture in a running fashion to pexy the small bowel mesentery to the ascending colon and proximal transverse colon mesentery. This was done medially to laterally along the mesentery in four different areas with the suture passing only through the thin peritoneal layer (Fig. 2B). Upon approximation, the small bowel mesentery appeared shortened and thickened, making it less prone to torsion (Fig. 2C). We returned the bowel into the abdominal cavity and covered it with omentum. We used #1 looped PDS to close the abdominal fascia and interrupted figure-of-eight #1 Vicryl to reinforce the fascia.

The patient’s hospital course was complicated by prolonged ileus requiring a 3-week stay post-operatively. She was discharged home with total parental nutrition. She had a fall at home 1 month following the operation, sustaining a hip fracture requiring intramedullary nailing, and had a brief stay at an acute rehabilitation facility. She was gradually transitioned to oral intake over the course of 2 months and TPN was discontinued. At her 3-month follow-up, she was tolerating a soft diet and feeling well, with regular bowel function and adequate pain control without narcotic pain medications.

## DISCUSSION

Midgut volvulus in adults is rare, with only about 120 case reports and an additional 200 patients in case series reported in the literature as of 2020 [1]. Previous case reports have reported recurrent midgut volvulus in patients with a history of childhood Ladd’s procedure [1], or an isolated incident of volvulus in elderly patients with no predisposing factors [2]. Cases in the elderly were managed with simple untwisting of the mesentery [2, 3]. Overall, it is exceedingly rare to encounter primary midgut volvulus in adult patients with neither evidence of intestinal malrotation nor mechanical causes of volvulus, such as jejunal diverticula [4]. One case report describes recurrent midgut volvulus in a patient with a diagnosis of Marfan’s syndrome in which the mesentery was noted intraoperatively ‘to appear long with a narrow base and floppy, likely contributing to hypermobility of the small bowel’ [5]. This description is strikingly similar to the appearance of the mesentery in our case (Fig. 2); however, our patient does not carry a diagnosis of Marfan’s, and the management in that case involved simple detorsion without mesenteric pexy. We present, to our knowledge, the only case of recurrent primary midgut volvulus in an adult with definitive management of small bowel mesentery pexy to the mesocolon as described in the operative discussion above. Another rare feature of our case is that simple detorsion was conducted in the first two laparotomies, which provided only a temporary solution. It was not until the third presentation and subsequent laparotomy that pexy was performed due to concern for the morbidity associated with repeated laparotomies in an elderly patient, along with that of ischemia associated with volvulus. It is also a possibility that our patient’s recent diagnosis of Crohn’s disease may have contributed to her presentation; however, there was no evidence of inflammatory changes along the bowel to suggest an acute flare. The primary difference in the post-operative course after pexy in our case was prolonged ileus. Consideration is given to the possibility that the mesenteric nerve plexus could have been disrupted by the pexy itself. However, the risk of temporary ileus likely is preferable in most cases to the need for reoperations for recurrent torsion if the mesentery is not pexied. We conclude that in the absence of high-level evidence in the form of controlled trials to support specific management of adult midgut volvulus, our technique is effective in preventing recurrent volvulus.

## CONCLUSION

Primary midgut volvulus in an adult is rare and can pose a challenge to surgeons, given that, in many instances, no definite source of volvulus can be identified. Previous case reports describe simple detorsion of the mesentery or performing duodenal and cecal pexy similar to a Ladd’s procedure. Our case report describes enteropexy of the small bowel mesentery to the mesocolon in the case of elongated small bowel mesentery causing recurrent volvulus. Our experience shows that this is an effective technique for preventing recurrent volvulus, especially in an elderly patient who may poorly tolerate repeated operations.
